# Real-world evaluation of deep learning decoders for motor imagery EEG-based BCIs

**DOI:** 10.3389/fnsys.2025.1718390

**Published:** 2025-12-15

**Authors:** Pierre Sedi Nzakuna, Emanuele D'Auria, Vincenzo Paciello, Vincenzo Gallo, Ernest Nlandu Kamavuako, Aimé Lay-Ekuakille, Kyandoghere Kyamakya

**Affiliations:** 1 Department of Industrial Engineering, University of Salerno, Fisciano, Italy; 2 Department of Engineering, King's College London, London, United Kingdom; 3 Faculty of Medicine, University of Kindu, Kindu, Democratic Republic of Congo; 4 Department of Engineering for Innovation, University of Salento, Lecce, Italy; 5 Department of Electronics, ISPT-Kin, Kinshasa, Democratic Republic of Congo; 6 Department of Smart System Technologies, University of Klagenfurt, Klagenfurt, Austria; 7 Department of Electrical and Computer Engineering, Polytechnic Faculty, University of Kinshasa, Kinshasa, Democratic Republic of Congo

**Keywords:** electroencephalography, brain-computer interfaces, motor imagery, deep learning, digital signal processing

## Abstract

**Introduction:**

Motor Imagery (MI) Electroencephalography (EEG)-based control in online Brain-Computer Interfaces requires decisions to be made within short temporal windows. However, the majority of published Deep Learning (DL) EEG decoders are developed and validated offline on public datasets using longer window lengths, leaving their real-time applicability unclear.

**Methods:**

To address this gap, we evaluate 10 representative DL decoders, including convolutional neural networks (CNNs), filter-bank CNNs, temporal convolutional networks (TCNs), and attention- and Transformer-based hybrids-under a soft real-time protocol using 2-s windows. We quantify performance using accuracy, sensitivity, precision, miss-as-neutral rate (MANR), false-alarm rate (FAR), information-transfer rate (ITR), and workload. To relate decoder behavior to physiological markers, we examine lateralization indices, mu-band power at C3 vs. C4, and topographical contrasts between MI and neutral conditions.

**Results:**

Results show shifts in performance ranking between offline and online BCI settings, along with a pronounced increase in inter-subject variability. Best online means were FBLight ConvNet 71.7% (±2.1) and EEG-TCNet 70.0% (±5.3), with attention/Transformer designs less stable. Errors were mainly Left-Right swaps while Neutral was comparatively stable. Lateralization indices/topomaps revealed subject-specific μ/β patterns consistent with class-wise precision/sensitivity.

**Discussion:**

Compact spectro-temporal CNN backbones combined with lightweight temporal context (such as TCNs or dilated convolutions) deliver more stable performance under short-time windows, whereas deeper attention and Transformer architectures are more susceptible to variation across subjects and sessions. This study establishes a reproducible benchmark and provides actionable guidance for designing and calibrating online-first EEG decoders that remain robust under real-world, short-time constraints.

## 1 Introduction

Brain-Computer Interfaces (BCIs) translate neural activity into control signals for communication and interaction, eliminating the need for muscular output. Non-invasive electroencephalography (EEG) remains the most widely used sensing modality due to its affordability, portability, and millisecond-scale temporal resolution, which enables closed-loop applications ranging from assistive communication to neurorehabilitation (Nicolas-Alonso and Gomez-Gil, 2012).

Motor imagery (MI) is the mental simulation of a physical motor action without actual movement, in which the subject imagines performing a motor task while remaining physically still (Lotze and Halsband, 2006). When a group of neurons is engaged in a task (like imagining movement), they stop their rhythmic, synchronized “idling” state and begin firing in a more complex, asynchronous pattern to handle the information. This chaotic firing leads to a measurable drop in signal power, known as Event-Related Desynchronization (ERD), which is therefore a marker of an active, processing cortex. For MI tasks, ERD is expected in the mu—similar to alpha but physiologically distinct (8–12 Hz)—and beta (12–30 Hz) brain sensorimotor rhythms over the sensorimotor cortex. Conversely, Event-Related Synchronization (ERS) is interpreted as a state of cortical idling or inhibition: neurons fire in a highly synchronized, rhythmic manner, producing a strong, high-power signal. It can occur in brain areas not involved in the MI task, or after an MI task is completed (Bansal and Mahajan, 2019; Pfurtscheller et al., 2006). Generally, contralateral ERD is expected in sensorimotor rhythms: stronger mu and/or beta ERD over the right hemisphere for left-hand MI and over the left hemisphere for right-hand MI. In both cases, ipsilateral ERS is expected over the same hemisphere as the imagined hand during and/or at the end of the MI task. However, noticeable intra- and inter-subject variability in the reactivity of mu-band components, such as ipsilateral or bilateral ERD during hand MI tasks, can still be observed in some subjects (Pfurtscheller et al., 2006; Nam et al., 2011; van Wijk et al., 2012; Hasegawa et al., 2017).

Offline BCIs are operated using preregistered datasets to develop and test preprocessing and decoding methods. In contrast, online BCIs operate on real-time, streamed data directly from the subject's head. In synchronous BCIs, the system determines when the user should issue a specific command, whereas in asynchronous BCIs, the user can issue commands at any time, making them more flexible and natural but more challenging to implement because the system must distinguish between intentional commands and idle brain activity. Exogenous BCIs use an external stimulus to generate a neural brain response. In contrast, endogenous BCIs are preferred for MI, as they allow the subject to generate the desired brain pattern internally, thereby mimicking natural motor control (Das et al., 2019; Bansal and Mahajan, 2019). Foundational surveys emphasize that dependable operation requires not only high accuracy but also responsiveness and stable user-system adaptation, typically summarized through information-transfer metrics and end-to-end latency constraints (Nicolas-Alonso and Gomez-Gil, 2012).

Over the past decade, deep learning (DL) has substantially advanced EEG decoding. Early convolutional neural networks (CNNs) demonstrated that end-to-end networks can learn discriminative spatiotemporal features directly from raw signals, providing interpretable representations of neurophysiological patterns and thereby merging feature extraction and machine learning stages in the former BCI design. Compact DL architectures further distilled these ideas into depth-wise separable designs tailored to EEG, facilitating deployment under resource constraints. More recently, hybrid convolution-attention models and transformer-based decoders have expanded temporal receptive fields and cross-channel context, reporting strong results in MI and other paradigms (Altaheri et al., 2023b; Stieger et al., 2021; Sedi Nzakuna et al., 2025b). However, evaluation practices remain heterogeneous across datasets and protocols.

Despite these advances, a gap persists between the validation of offline simulations and the demands of real-world interactive rapid control. Many model-centric studies develop and assess novel decoders on offline BCI datasets using multi-second windows that exceed the temporal budget of real-time rapid BCI operation. In practice, decisions must be issued within brief windows under tight latency budgets, which reduces the available evidence per decision and can depress accuracy, increase false positives, and destabilize calibration. Prior work on time-window effects supports this concern, showing that shortening the window often degrades performance, even when decoders are well tuned, highlighting the need for systematic evaluation under short-window constraints (Miladinovic et al., 2024; Mart́ın-Chinea et al., 2023).

This paper addresses the deployment gap by evaluating representative DL EEG decoders from the literature under a soft real-time streaming setup (online trial-wise prediction < 1 s). We contrast reported performance across longer analysis windows and brief, rapid, and real-time-feasible time windows in an online BCI paradigm while enforcing strict latency budgets. We report classification accuracy, sensitivity, precision, F1 score, miss-as-neutral rate, false alarm rate, information transfer rate, and mental workload. In addition, we analyze architectural and training strategies that mitigate short-window degradation.

The contributions of this study can be summarized as follows:

- A reproducible evaluation protocol for DL EEG decoders that operationalizes real-world timing and decision constraints for online MI BCI.
- A cross-metric assessment of online effectiveness and efficiency of DL decoders, including accuracy, sensitivity, precision, miss-as-neutral (MANR), false-alarm rate (FAR), information transfer rate (ITR), and workload during brief decision windows.
- A broad, architecture-spanning comparison of ten representative models covering shallow and spatial CNNs, filter-bank CNNs, temporal-convolutional backbones, attention, and Transformer hybrids under a unified training and evaluation pipeline.
- Neurophysiology-informed analyses that explain subject-specific error modes (MANR and FAR) and ranking shifts in the outcomes of DL decoders in an online BCI setting.
- Actionable guidance for online-first DL decoder design and calibration, aimed at improving stability and ITR without increasing latency.

The remainder of this work is organized as follows. Section 2 presents the materials and methods used for this study, including the DL EEG decoders under study. Section 3 describes the setup of our experiment. Section 4 presents the results and discusses them, followed by practical recommendations in Section 5. Section 6 states the limitations of this study and provides useful indications for future work. Finally, the Conclusion summarizes the study's outcomes.

## 2 Materials and methods

### 2.1 Subjects and EEG signal acquisition

Four healthy young male participants, aged 20–28, with no record of neurological illness, participated in our experiment. Only the fourth subject had previously participated in an MI online BCI experiment. The third participant was left-handed, while the other participants were right-handed.

**Figure 1:**
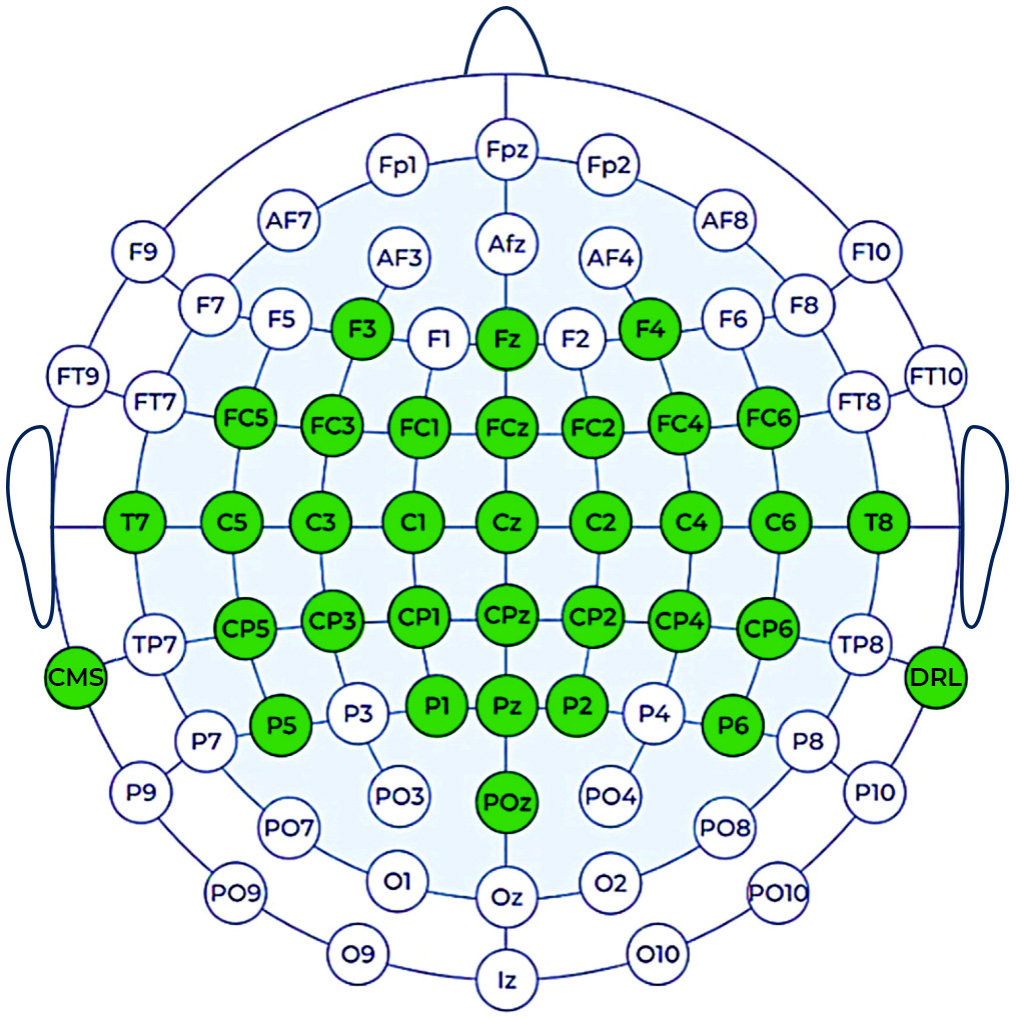
Selected electrode channel locations on the Emotiv Flex Gel helmet.

In this study, we used the Emotiv Flex Gel noninvasive neuro-helmet for EEG signal acquisition. We used the following 32 electrode channels, in accordance with the 10–20 positioning system: Cz, FCz, Fz, F3, FC1, C1, C3, FC3, FC5, T7, C5, CP5, CP3, CP1, P1, P5, CPz, Pz, POz, P6, P2, CP2, CP4, CP6, C6, T8, FC6, FC4, C4, C2, FC2, and F4. This electrode montage is sensorimotor-centric, with premotor, Supplementary Motor Area (SMA), and dorsal prefrontal support, as well as posterior parietal and light temporal coverage (Strotzer, 2009; Baars and Gage, 2010). This represents a solid MI montage with good spatial coverage of the pre-motor and motor cortex regions over the scalp, which are responsible for physical motion intention and control; therefore, it is appropriate for our MI experiment, as in (Kwon et al. 2020). The channel location TP9 served as a Common Mode Sense (CMS) reference, while the channel location TP10 was used as a Driven Right Leg (DRL). Figure 1 shows the montage of electrode channel locations we used for this study.

EEG signals were recorded at a sampling rate of 256 samples per second. The analog microvoltages measured across participants' scalps were internally converted to digital values by the helmet's dual 16-bit ADCs, with 1 LSB corresponding to 0.51 μ*V* and two bits of instrumental noise floor discarded. The EEG data were then streamed in real time to the personal computer (PC) via a Bluetooth 5.1 connection between the helmet and its paired USB dongle, which was inserted into the PC. To ensure high-quality EEG acquisition, we continuously monitored signal quality on the PC using the Emotiv software suite.

### 2.2 EEG signal preprocessing

The helmet has an internal bandwidth ranging from 0.16 to 43 Hz, with digital notch filters at 50 and 60Hz. We applied additional bandpass filtering on the PC side, selecting a frequency range of 2–29 Hz for all participants using a fourth-order Butterworth filter. The selected frequency range includes the upper half of the delta (0.5–4 Hz), the entire theta (4–8 Hz), alpha with mu (8–12 Hz), and beta (12–29 Hz) sensorimotor brain rhythms, without touching the 30 Hz upper bound to avoid overlap with the gamma rhythms. The lower bandpass cutoff was experimentally set to 2 Hz, as confirmed by recent studies, which demonstrated that this can improve classification accuracy when using DL EEG decoders (Kessler et al., 2025; Wei et al., 2019). There was no power-line interference to consider, as the helmet was powered by its internal LiPo battery.

### 2.3 Deep learning architectures and datasets

To make our comparison meaningful and forward-looking, we selected 10 DL architectures tailored for MI EEG decoding from the Brain Decode 0.8.1 library (Schirrmeister et al., 2017), which contains the most recent state-of-the-art efficient DL architectures for EEG decoding. The selected decoders cover the major architectural design assumptions used in MI EEG decoding over the last seven years: classic shallow CNNs rooted in FBCSP principles (Shallow FBCSP Net, EEGNet), spatially focused CNNs (SCCNet), temporal-convolutional backbones for short-window dynamics (EEG-TCNet), filter-bank architectures that explicitly model sub-bands (FBCNet, FBLight ConvNet), cross-frequency and interaction modeling (IFNet), and attention and Transformer families that capture longer-range dependencies (EEG Conformer, Attention BaseNet, MSVTNet). Table 1 summarizes the selected DL architectures in this study—listed in descending order by year of release, the preprocessing methods applied to EEG data, the test configuration, and the reported decoder accuracy. In this study, we used these models with their default parameters from the Brain Decode library.

**Table 1:** Selected deep learning architectures for motor imagery EEG decoding.

**#**	**Model (year)**	**Description**	**Data preprocessing**	**MI dataset test configuration and reported accuracy %**
1	MSVTNet (Liu et al., 2024) (2024)	Multi-Scale Vision Transformer Neural Network (MSVTNet) extracts spatio-temporal features at various filtered scales via CNNs, merges them into multi-scale tokens, and uses Transformers to model cross-scale interactions and global temporal patterns. An auxiliary branch loss aids intermediate supervision for effective CNN-Transformer integration.	*Z*-score normalization for the BCIC-IV 2a and 2b. A fifth-order Butterworth bandpass filter from 4 to 30 Hz for OpenBMI. One-fold data augmentation by segmentation and reconstruction.	•BCIC-IV 2a: 4s, 250 Hz, 1,000 samples, 22 channels, HO, 82.56% •BCIC-IV 2b: 4s, 250 Hz, 1,000 samples, 3 channels, 5-fold CV, 70.30% •OpenBMI: 4s, 250 Hz, 1,000 samples, 20 channels, HO, 75.93%
2	Attention BaseNet (Wimpff et al., 2024) (2024)	AttentionBaseNet is a convolution-first EEG model with a channel-attention block. It extracts temporal and spatial features, expands channels via 1 × 1 convolution, refines timing with depthwise–pointwise convolutions, and applies optional attention.	40 Hz low-pass filtering for BCIC-IV datasets, and a 4 Hz high-pass filter is applied for the HGD.	•BCIC-IV 2a: 4s, 250 Hz, 1,000 samples, 22 channels, HO, 76.91% •BCIC-IV 2b: 4s, 250 Hz, 1,000 samples, 3 channels, HO, 85.32% •HGD: 4s, 250 Hz, 1,000 samples, 44 channels, HO, 94.88%
3	FBLight ConvNet (Ma et al., 2023) (2023)	Filter Bank Light Convolutional Network (FBLightConvNet) extracts spatial-spectral features from multi-view EEG, segments data into time windows, and captures MI patterns across stages. A temporal attention module assigns weights to each window to fuse features into a more discriminative representation.	Filter-bank decomposition: 9 bandpass Chebyshev-II filters of 4 Hz each from 4 to 40 Hz with 2 Hz transition bandwidth and 30 dB stopband ripple.	•BCIC-IV 2a: 4s, 250 Hz, 1,000 samples, 22 channels, HO, 79.48% •OpenBMI: 4s, 250 Hz, 1,000 samples, 20 channels, HO, 70.43%
4	IFNet (Wang et al., 2023) (2023)	Interactive Frequency Convolutional Neural Network (IFNet) that models cross-frequency MI interactions. It extracts spectro-spatial features from low- and high-frequency bands, combines them via element-wise addition and temporal pooling, and uses trial augmentation for final classification.	Downsampling from 250 Hz to 125 Hz, and bandpass-filtering at 0.5–38 Hz.	•BCIC-IV 2a: 4s, 250 Hz, 1,000 samples, 22 channels, HO, 78.21% •OpenBMI: 4s, 250 Hz, 1,000 samples, 20 channels, HO, 71.23%
5	EEG Conformer (Song et al., 2023) (2022)	EEG Conformer merges CNN and Transformer for EEG decoding. It extracts local features via temporal and spatial convolutions, then captures global temporal dependencies using self-attention. Final predictions are made using fully connected layers.	Segmentation and reconstruction in the time domain. Bandpass filtering between 4 and 40 Hz.	•BCIC-IV 2a: 4s, 250 Hz, 1,000 samples, 22 channels, HO, 78.66% •BCIC-IV 2b: 4s, 250 Hz, 1,000 samples, 3 channels, HO, 84.63%
6	FBCNet (Mane et al., 2021) (2021)	Filter-Bank Convolutional Network (FBCNet) for MI decoding through multi-view data representation, followed by spatial filtering to extract spectro-spatial features. This multistage approach enables efficient model training with limited data. A variance layer aggregates the EEG time-domain information.	Filter-bank decomposition: nine bandpass Chebyshev-II filters of 4 Hz each from 4 to 40 Hz with 2 Hz transition bandwidth and 30 dB stopband ripple.	•BCIC-IV 2a: 4s, 250 Hz, 1,000 samples, 22 channels, HO, 76.20% •OpenBMI: 4s, 250 Hz, 1,000 samples, 20 channels, 10-fold CV, 67.19% •Stroke A: 4s, 250 Hz, 1,000 samples, 27 channels, 10-fold CV, 79.16% •Stroke B: 4s, 250 Hz, 1,000 samples, 27 channels, 10-fold CV, 81.11%
7	EEG-TCNet (Ingolfsson et al., 2020) (2020)	EEG Temporal Convolutional Net (EEG-TCNet) is a lightweight temporal CNN built on top of EEGNet, aimed at offering high accuracy with minimal trainable parameters to suit edge devices.	Standardization through the removal of the mean and scaling to unit variance per channel.	BCIC-IV 2a: 4.5s, 250 Hz, 1125 samples, 22 channels, HO, 77.35%
8	SCCNet (Wei et al., 2019) (2019)	Spatial Component-wise Convolutional Network (SCCNet) for MI EEG decoding using an initial convolution layer for spatial filtering to extract components from multi-channel time-series EEG, with a lightweight design to prevent overfitting.	Downsampling from 250 Hz to 125 Hz, and bandpass-filtering at 0.5–38 Hz.	BCIC-IV 2a: 4.5s, 125 Hz, 562 samples, 22 channels, HO, 74.11%
9	EEGNet (Lawhern et al., 2018) (2018)	Compact CNN for EEG decoding that borrows depthwise and separable convolutions from computer vision to create an EEG-specific architecture incorporating well-known EEG feature extraction principles, while reducing the number of trainable parameters.	4–40 Hz third-order Butterworth bandpass filtering and exponential moving average window with a decay factor of 0.999.	BCIC-IV 2a: 4s, 250 Hz, 1,000 samples, 22 channels, 4-fold CV, 68%
10	Shallow FBCSP Net (Schirrmeister et al., 2017) (2017)	The Shallow ConvNet is a lightweight model inspired by FBCSP methods. It uses temporal and spatial convolutions to extract frequency-specific features, followed by squaring and logarithmic operations for non-linear transformation.	A third-order Butterworth filter is applied between 4 and 38 Hz for the BCIC-IV 2a and between 4 and 125 Hz.	•BCIC-IV 2a: 3.5s, 250 Hz, 875 samples, 22 channels, –, 71.9% •HGD: 4s, 250 Hz, 1,000, 44 channels, –, 93.9%

In addition to these selected models, we consider the following 10 models as additional offline baselines: TCANet (2025) (Zhao et al., 2025a), MSCFormer (2025) (Zhao et al., 2025b), CTNet (2024) (Zhao et al., 2024), EEGNeX (2024) (Chen et al., 2024), EEG SimpleConv (2023) (Ouahidi et al., 2023), EEG-ITNet (2022) (Salami et al., 2022), ATCNet (2022) (Altaheri et al., 2023a), EEG-inception (2021) (Zhang et al., 2021), TIDNet (2020) (Kostas and Rudzicz, 2020), and Deep ConvNet (2017) (Schirrmeister et al., 2017).

We constrained the online experiment evaluation to 10 representative decoders to preserve internal validity and participant welfare, since online MI testing entails sustained attention and repeated imagery: beyond a modest number of models, fatigue, habituation, and co-adaptation alter mu and beta bands ERD patterns, confounding between-architecture comparisons. Limiting the online set keeps session duration within acceptable bounds, enables counterbalanced ordering and stable thresholds, and yields cleaner within-subject estimates under short time windows. The broader architectural landscape is still covered via the additional offline baselines.

For selected models as well as additional models, the training was conducted using an Adam optimizer at a learning rate of 0.001, a batch size of 64, and 500 epochs.

In their featured papers, these DL architectures had been trained and validated using the following datasets in hold-out (HO) or *k*-fold cross-validation (CV) testing settings:

#### 2.3.1 BCI competition IV 2a (BCIC-IV 2a)

MI EEG data were collected using 22 electrodes at 250 Hz from nine healthy participants across two sessions on two different days (Brunner et al., 2008). Participants performed 72 trials per session for each of the following four MI mental tasks: left hand, right hand, feet, and tongue. Each trial lasted 4 s, comprising 1 s for cue display and 3 s for the MI task. Signals were band-pass filtered between 0.5 and 100 Hz, with an additional 50 Hz notch filter to remove line noise. The amplifier sensitivity was set to 100 μ*V*.

#### 2.3.2 BCI competition IV 2b

MI EEG data were recorded from nine right-handed participants using three electrodes (C3, Cz, and C4) across five sessions on two different days (Leeb et al., 2008). The first two sessions were feedback-free, while the last three had visual feedback. Signals were recorded at 250 Hz with a sensitivity of ±100μ*V*, then bandpass-filtered between 0.5 and 100 Hz, with a 50 Hz notch filter enabled. Participants performed 10 trials per class per run across the six runs per session during the two feedback-free sessions. This resulted in 120 trials per session for each of the two classes, namely the left-hand and right-hand MIs. Each trial lasted 8.5 s, including 4 s of mental task performance for the MI.

#### 2.3.3 OpenBMI

MI EEG data from 54 healthy participants, collected over two sessions on different days using 64 electrodes at 1,000 Hz (Lee et al., 2019). Each subject performed 100 trials of a 4-s MI task per session per class, for left- and right-hand classes.

#### 2.3.4 Stroke A

Post-stroke motor-rehabilitation EEG data from 37 patients who were asked to control an MI BCI (Ang et al., 2015). Two classes were recorded—the imagined movement of the stroke-paralyzed hand and the resting state—with 80 trials of 4 s each per class. EEG was recorded across 27 channels at 250 Hz and bandpass filtered between 0.5 and 40 Hz.

#### 2.3.5 Stroke B

Similar to the Stroke A dataset, but with 34 patients instead (Ang et al., 2014). Each subject underwent two runs; each run consisted of 40 MI trials for the stroke-impaired hand and 40 trials for the idle state, with each trial lasting 4 s. EEG signals were recorded across 27 channels at 250 Hz using the Nuamps EEG acquisition hardware. Signals were acquired with a resolution of 22 bits and a sensitivity of ±130 mV, then band-pass filtered between 0.5 and 40 Hz.

#### 2.3.6 High-gamma dataset (HGD)

EEG data were recorded from 14 healthy participants using 128 electrodes placed over the motor cortex. For each of the four classes—clenching toes, left-hand fingers, right-hand fingers, and idle—the participants performed 20 trials per run across 13 runs, totaling 260 trials of 4 s each per class (Schirrmeister et al., 2017).

### 2.4 Evaluation metrics

To ensure the readiness of an online BCI for use in real-world scenarios, its evaluation must consider the following three aspects (Pan et al., 2024):

- The usability, defined as the effectiveness (ability to produce correct and reliable results) and efficiency (speed and cognitive resources required from the user), with which a user achieves their set objectives.
- The user satisfaction, which investigates the subjective perception of both the general aspects of assistive technology and the specific characteristics of the BCI.
- Usage is understood as the degree of correspondence between the system's decisions and the user's intentions in everyday use.

#### 2.4.1 Effectiveness

This quantifies the system's capacity to achieve correct and trustworthy outcomes.

- Accuracy measures the proportion of model predictions that match the true data labels out of all model predictions in both offline and online experiments. Values closer to 1 indicate better performance.

$$\begin{array}{l} \text{Accuracy}=\frac{\text{Number of correct model predictions}}{\text{Total number of model predictions}} \end{array}$$ (1)

In MI EEG applications, 70% is generally considered the minimum acceptable accuracy threshold; any value below this threshold is interpreted as BCI illiteracy (Sedi Nzakuna et al., 2025a).

- A confusion matrix shows the distribution of predictions across all classes, revealing which classes are misclassified and the types of errors made. Accuracy alone can hide poor performance on specific classes, especially in imbalanced datasets. The confusion matrix exposes these weaknesses by displaying true positives (TP), false positives (FP), false negatives (FN), and true negatives (TN) for each class, enabling a deeper understanding of model performance.
- Sensitivity, also known as the True Positive Rate (TPR) or Recall, measures how well the model identifies actual positives within each class. High sensitivity means the model rarely misses positive cases.

$$\begin{array}{l} \text{Sensitivity}=\frac{\text{True Positives}}{\text{True Positives + False Negatives}} \end{array}$$ (2)

- Precision measures how many predicted positives are actually correct. High precision means few false alarms.

$$\begin{array}{l} \text{Precision}=\frac{\text{True Positives}}{\text{True Positives + False Positives}} \end{array}$$ (3)

- The Miss-as-Neutral Rate (MANR) indicates, in online BCIs, the number of trials from other classes that are misclassified as trials of the Neutral (idle) class. A high value means reduced usability of the BCI for the user.
- False Alarm Rate (FAR) indicates, in online BCIs, the number of times the BCI misclassifies the user's neutral state as an intention of other classes. A low value indicates safer BCI control, with the BCI responding only when the user's intention is to target a class other than neutral.

#### 2.4.2 Efficiency

This quantifies how quickly and easily users communicate their intentions to the BCI online, evaluating both the speed of command transmission and the cognitive workload required.

- Information transfer rate (ITR) quantifies the amount of information (in bits) communicated by the user per unit of time (usually minutes), accounting for both the accuracy and speed of the BCI. Higher ITR means faster and more accurate communication. If accuracy is low, ITR drops significantly even if speed is high.

$$\begin{array}{l} \text{ITR}=\frac{60}{T}\left[\log_{2}N+P\log_{2}P+\left(1-P\right)\log_{2}\left(\frac{1-P}{N-1}\right)\right] \end{array}$$ (4)

where *N* denotes the number of possible target classes, *P* the accuracy, and *T* the time (in seconds) required to output a command.

- Mental workload refers to the cognitive load required from the user to perform BCI tasks effectively. It is influenced by factors such as the user's psychological state, experience with BCI operation, and task complexity. The lower the mental workload, the better the user's experience. The NASA Task Load Index (NASA-TLX) scale is widely used to evaluate the mental workload of users operating a BCI, using the following six parameters: mental demand, physical demand, temporal demand, effort level, performance level, and frustration level (Hart, 2006; Pan et al., 2024). Each parameter receives a score between 0 and 100: 0–10 indicates very low effort, 10–35 indicates the desired level of effort, 35–65 indicates moderate effort, 65–90 indicates excessive effort, and 90–100 indicates very high effort. High scores indicate excessive effort, an ineffective interface, and slow or difficult use and should be optimized.

### 2.5 Spectral analysis of EEG data

To relate subject-specific neurophysiologic behavior to online decoding, we compute the following four spectral descriptors in the μ-band range.

#### 2.5.1 Lateralization Index (LI) as a function of frequency

For each trial, we estimate the power spectral density (PSD) without overlap across narrow 1 Hz bands from 2 to 29 Hz. Let *P*_*C*3_(*f*) and *P*_*C*4_(*f*) be the band power at C3 and C4, respectively. The LI is defined per trial and band as follows:


$$\begin{array}{l} \text{LI}\left(f\right)=\frac{P_{C4}\left(f\right)-P_{C3}\left(f\right)}{P_{C4}\left(f\right)+P_{C3}\left(f\right)} \end{array}$$ (5)

Class-wise curves were obtained by averaging LI across trials belonging to the same class. Higher positive LI values indicate relatively stronger right-hemisphere activity (or weaker C3 power), while more negative values indicate relatively stronger left-hemisphere activity (or weaker C4 power).

#### 2.5.2 C3 vs. C4 Mu-band power

To visualize contralateral desynchronization at a glance in a scatter plot, we compute the PSD from 8 to 12 Hz at the C3 and C4 electrode locations using Welch's method and plot the per-trial results. This highlights quadrant separation relative to the identity line. A clean MI signature would appear as class-biased quadrants: left MI tending toward higher C3 than C4 (points below the diagonal) and right MI tending toward higher C4 than C3 (points above the diagonal), with neutral clustered near the diagonal and at lower radii.

#### 2.5.3 ERD/ERS topographic maps analysis

To quantify cortical activation patterns during MI, we computed ERD and ERS in specific frequency bands. The Neutral class served as a baseline reference for calculating percentage changes in band power during MI tasks.

For each trial, we estimated PSD using Welch's method (512-point FFT, 50% overlap) in three frequency bands of interest: theta rhythm (4–8 Hz), mu rhythm (8–12 Hz), and beta rhythm (13–29 Hz). The percentage change in band power for each MI condition (left and right limbs) relative to the neutral baseline is computed as follows:


$$\begin{array}{l} \text{Power Change (\%)}=\frac{P_{\text{MI}}-P_{\text{Neutral}}}{P_{\text{Neutral}}}\times 100 \end{array}$$ (6)

where *P*_MI_ represents the average band power during MI trials, and *P*_Neutral_ represents the average band power during Neutral trials. Negative values indicate ERD (decreased power, reflecting cortical activation), while positive values indicate ERS (increased power, reflecting cortical idling or inhibition).

Topographical maps are generated to visualize the spatial distribution of these percentage changes across the 32-electrode montage, facilitating the identification of contralateral activation patterns characteristic of MI. The topographical maps use a red-white-blue color scheme: red tones indicate ERS (power increase, i.e., cortical idling), blue tones indicate ERD (power decrease, i.e., cortical activation), and white indicates no change from baseline. This visualization enables immediate identification of cortical activation patterns during MI tasks.

## 3 Experiment setup

### 3.1 Environment

**Figure 2:**
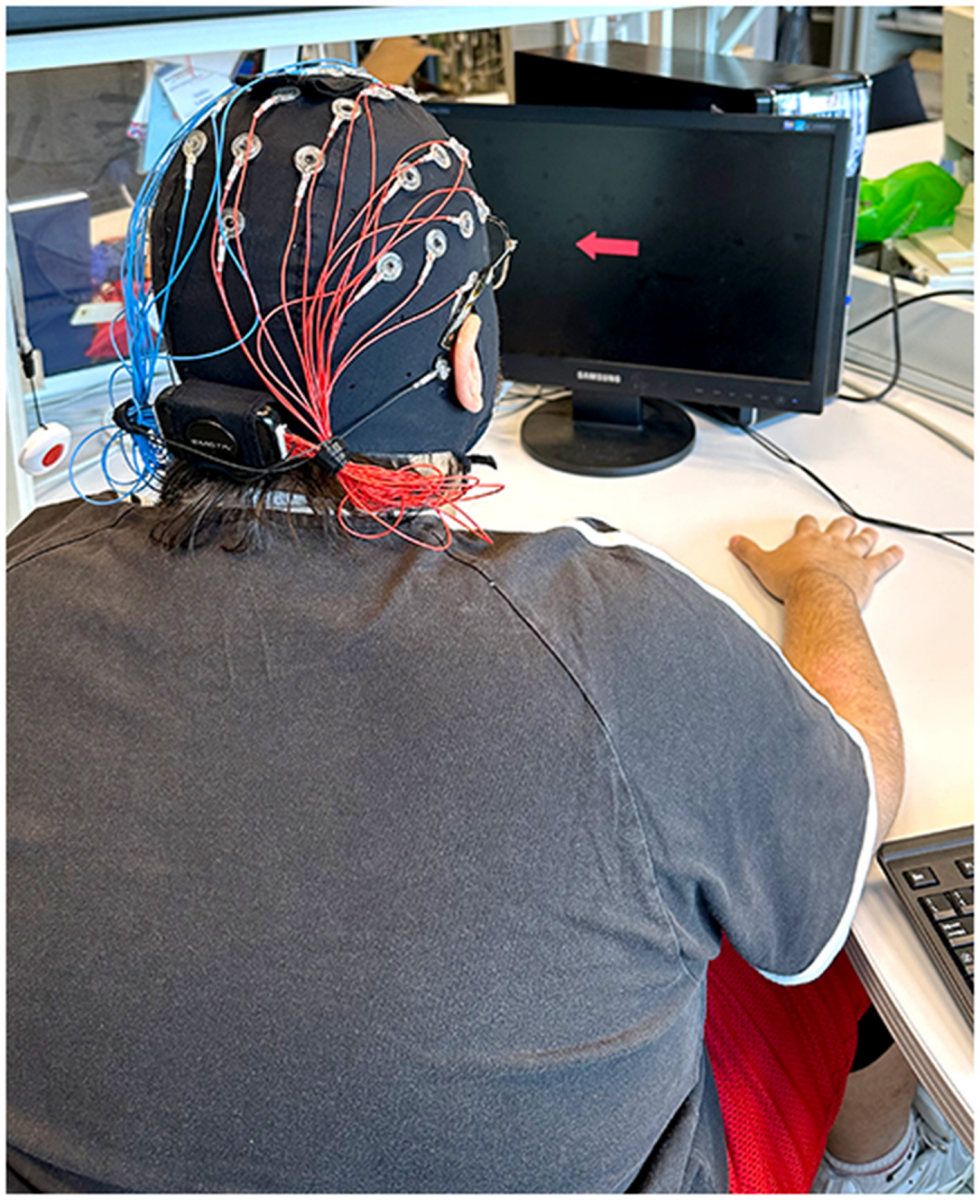
Subject 2 wearing the Emotiv Flex Gel helmet and seated in front of the PC screen during the BCI experiment.

We conducted our experiment in the Laboratory of Measurements and Instrumentation at the University of Salerno under calm conditions that allowed for optimal participant concentration. Participants were seated on a chair in front of a PC screen, as shown in Figure 2, and were instructed to relax and avoid voluntary head or body movements during the idle state in both the offline and online BCI sessions. Participants were informed about the goals of our study and were invited to sign a consent form before starting the experiment.

EEG signals acquired using the Emotiv Flex Gel helmet were continuously streamed via a Bluetooth link to the USB dongle connected to the PC. The PC was equipped with an Intel Core i7-930 CPU, 12 GB of RAM, an NVIDIA GeForce RTX 2080 GPU with 8 GB of VRAM, and it was running Windows 10.

### 3.2 Experiment protocol

#### 3.2.1 Offline BCI session

First, each participant underwent a data registration session for model training. Each trial lasted 8 s in total, including 0.5 s for displaying the red cue on the screen—left and right arrows indicating the left and right classes, respectively—1.5 s with a black screen, 2 s for the participant to perform the requested mental MI task with a white fixation cross displayed on the screen, and 4 s for resting with a black screen. For the Left class, participants were instructed to perform mental MI of their left limbs (i.e., left hand and left foot), while the Right class performed mental MI of their right limbs. The idle state was considered the Neutral class, with no cue or fixation cross displayed on the screen during EEG recording.

**Figure 3:**
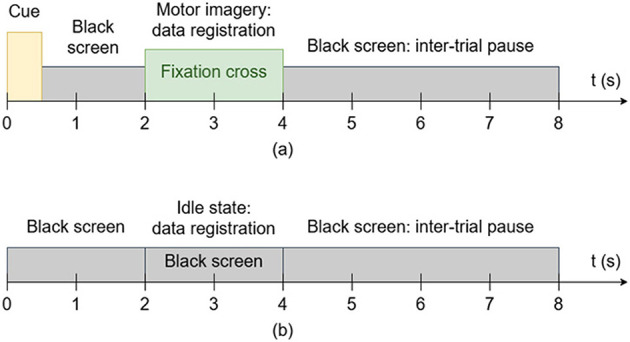
Timing scheme of the data registration session for: **(a)** Left and right classes, **(b)** neutral class.

The registration session was split into five subsessions. Each subsession consisted of 15 trials for each of the three classes, for a total of 75 per class across the entire registration session. Figure 3 shows the timing scheme of the data registration session. EEG signals were acquired at a sampling frequency of 256 Hz for 2 s, resulting in 512-time samples per trial. We conducted model training and validation using a stratified four-fold CV.

#### 3.2.2 Online BCI session

**Figure 4:**
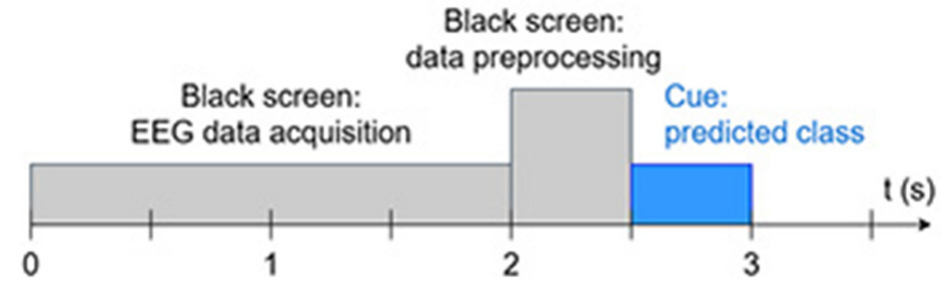
Timing scheme of the online BCI session.

In the online BCI session, participants were asked to operate the BCI in an asynchronous endogenous control mode. During the testing of each of the selected DL decoders, the BCI experiment operator randomly instructed participants to perform a total of 15 left-class and 15 right-class MI trials, each lasting at least 3 s, with the computer recognizing the mental MI activity and displaying on the screen the cue (left or right arrow) corresponding to the predicted class. No cue was displayed on the screen when the Neutral class was predicted. The time window was set to 2 s at a 256 Hz sampling frequency (i.e., 512 time samples), and the PC was expected to preprocess the acquired signal and predict the correct class within 1 s before resuming EEG data acquisition. Figure 4 shows the timing scheme of the online BCI session.

If, after 10 s—that is, three time frames of 3 s each (Figure 4)—the BCI system failed to recognize the mental MI activity performed by the participant as instructed by the experiment operator, the latter marked a Miss-as-Neutral for the requested class other than Neutral. Conversely, if the BCI predicted either the left or right class and displayed the corresponding arrow on the screen while the participant was in an idle state, the operator marked this event as a false alarm. We experimentally set the thresholds for the left and right classes to 40%, given the overlap of lateralized rhythms during active segments relative to the idle state.

## 4 Results and discussion

### 4.1 Effectiveness

**Figure 5:**
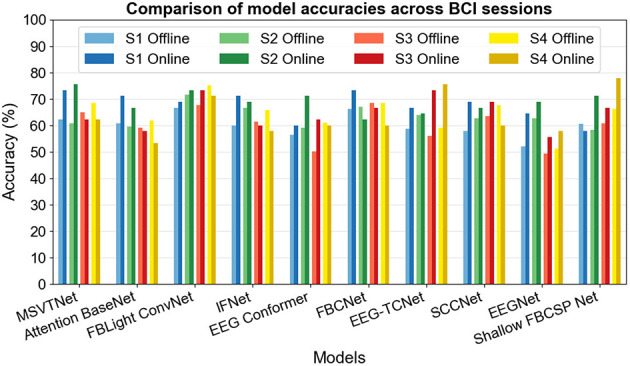
Comparison of selected model accuracies across BCI sessions.

Table 2 reports the offline and online accuracies of the DL EEG decoders tested in this study, and Figure 5 presents a visual comparison of these offline and online classification accuracies for each decoder. We observe in Table 2 that almost all models yielded better performance in the online session for Subjects 1 and 2, while results are more balanced for Subject 3 and worse in the online session for Subject 4. These results show that offline rankings do not reliably predict interactive performance. Several architectures improve when moved to the streaming setting (e.g., EEG-TCNet, MSVTNet, Shallow FBCSP Net, EEG Conformer), while others lose ground (e.g., FBCNet for multiple subjects). Among the strongest online methods are the compact spectro-temporal CNNs and temporal convolutional designs (FBLight ConvNet and EEG-TCNet), with MSVTNet close behind. The shallow FBCSP baseline is also competitive online, but with a higher subject-to-subject spread. From Figure 5, it can be seen that, overall, FBLight ConvNet exhibits more stable online performance around the 70% accuracy threshold, with the smallest dispersion across subjects, suggesting a conservative inductive bias that generalizes well to short-window, latency-constrained inference. In contrast, decoders with larger capacity or heavier attention appear more sensitive to subject shift and session dynamics, with pronounced accuracy gaps between offline and online BCI sessions and classification accuracy around 60%. Overall, on average, only FBLight ConvNet and EEG-TCNet reached the 70% accuracy threshold in online BCI operation.

**Table 2:** Accuracy (%) of the selected DL EEG decoders in both Offline and Online BCI sessions.

**#**	**Model**	**Subject 1**		**Subject 2**		**Subject 3**		**Subject 4**		**Mean** ±**SD**	
		**Offline**	**Online**	**Offline**	**Online**	**Offline**	**Online**	**Offline**	**Online**	**Offline**	**Online**
1	MSVTNet	62.23	**73.33**	60.87	**75.56**	**64.89**	62.22	**68.45**	62.22	64.11 ±**3.34**	**68.33** ± 7.12
2	Attention BaseNet	60.87	**71.11**	59.54	**66.67**	**59.14**	57.78	**61.80**	53.33	60.33 ±**1.22**	**62.22** ± 8.12
3	FBLight ConvNet	66.63	**68.89**	71.55	**73.33**	67.58	**73.33**	**75.16**	71.11	70.23 ± 3.92	**71.67** **±2.13**
4	IFNet	60.00	**71.11**	66.69	**68.89**	**61.32**	60.00	**65.80**	57.78	63.45 ±**3.29**	**64.45** ± 6.54
5	EEG conformer	56.46	**60.00**	59.09	**71.11**	50.21	**62.22**	**60.96**	60.00	56.68 ±**4.69**	**63.33** ± 5.29
6	FBCNet	66.27	**73.33**	**67.11**	62.22	**68.46**	66.67	**68.44**	60.00	**67.57** **±1.07**	65.55 ± 5.88
7	EEG-TCNet	58.66	**66.67**	63.97	**64.44**	55.98	**73.33**	59.18	**75.56**	59.45 ±**3.33**	**70.00** ± 5.29
8	SCCNet	57.76	**68.89**	62.65	**66.67**	63.53	**68.89**	**67.55**	60.00	62.87 ±**4.02**	**66.11** ± 4.21
9	EEGNet	51.96	**64.44**	62.68	**68.89**	49.33	**55.56**	51.12	**57.78**	53.77 ±**6.04**	**61.67** ± 6.12
10	Shallow FBCSP Net	**60.47**	57.78	58.22	**71.11**	60.88	**66.67**	66.24	**77.78**	61.45 ±**3.40**	**68.34** ± 8.39

Bold values indicate the best results.

However, the online protocol used in this study should be considered when interpreting the results, as each online trial allowed up to three decoding attempts within a 10-s window. This closed-loop, repeated-attempt design provided participants with the opportunity to retry until they received visual feedback, so the final cue displayed online was not always a single-shot decision made within a fixed window but rather the best of a sequence of attempts in some cases. Consequently, a higher online average may partly reflect human—in-the-loop correction, model confidence, or latency behavior, and interface responsiveness rather than purely static classifier quality. In other words, offline-online gaps should be interpreted as differences in system-level performance under interaction, not simply as model drift. The average time required by the computer to perform signal preprocessing and trial-wise prediction during the online BCI session did not exceed 0.6 s, i.e., effectively less than 1 s, as expected from our soft real-time paradigm.

**Figure 6:**
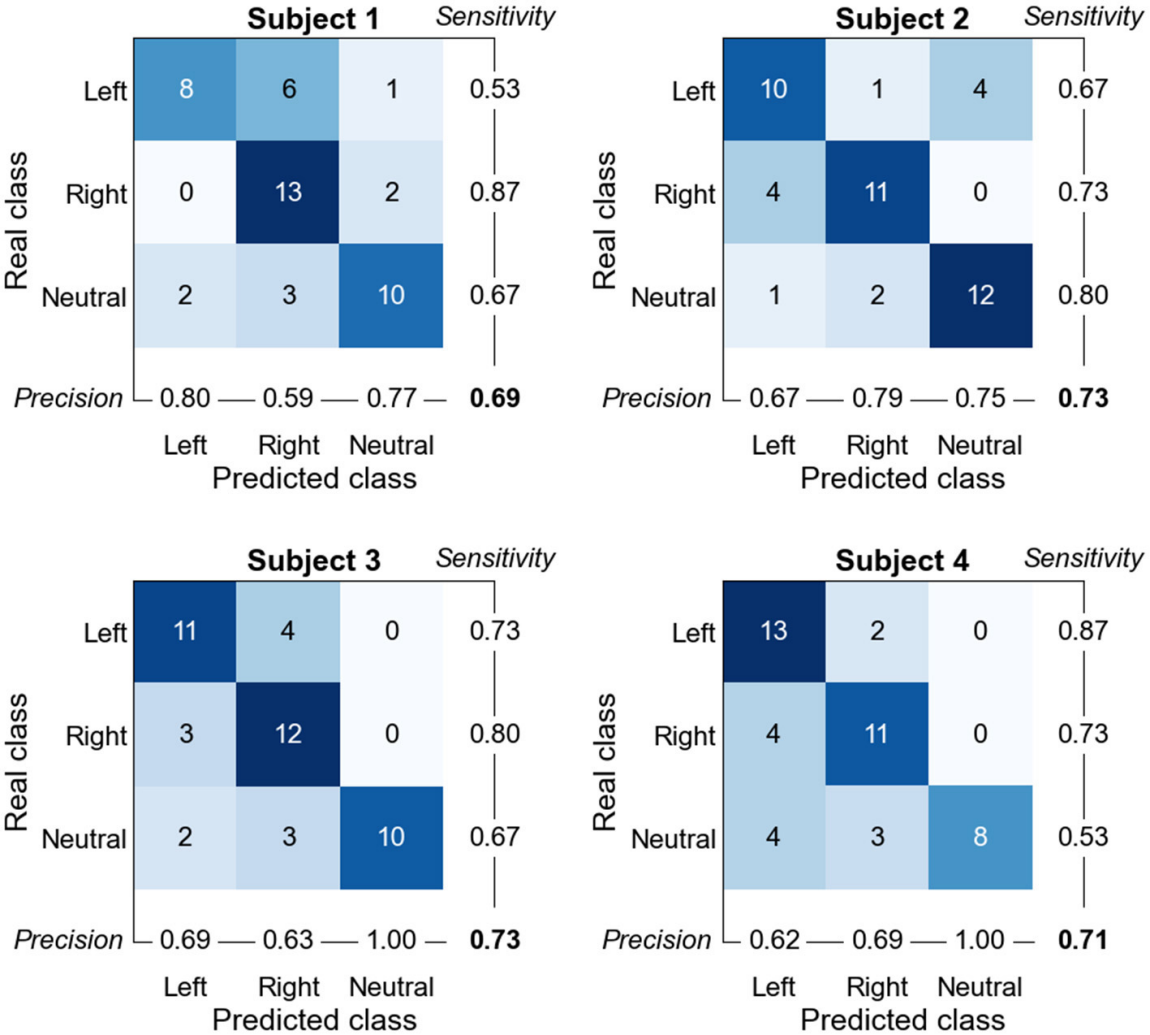
Online Confusion matrices across Subjects for FBLight ConvNet.

Confusion matrices of all four participants for FBLight ConvNet are shown in Figure 6. We observe a consistent left-right interchange as the dominant misclassification error, while the neutral class tends to be preserved—an expected pattern for MI where lateralized rhythms overlap while rest segments are more stationary. The asymmetry of mistakes across subjects (e.g., one participant showing more left-right confusions, another more neutral-left), as well as the observed mid-to-high range sensitivity (per subject, per class), with the weakest values typically on one lateral class for a given participant (e.g., left for one subject, neutral for another), points to individual differences rather than a single systematic failure. These observations suggest concrete improvements: emphasize lateralized features over central channels (e.g., stronger spatial filtering around C3/C4), incorporate temporal smoothing or selective abstention to curb momentary flips between left and right, and use brief per-user calibration with lightweight adaptation (e.g., temperature scaling or class-wise thresholding) to correct subject-specific biases.

**Figure 7:**
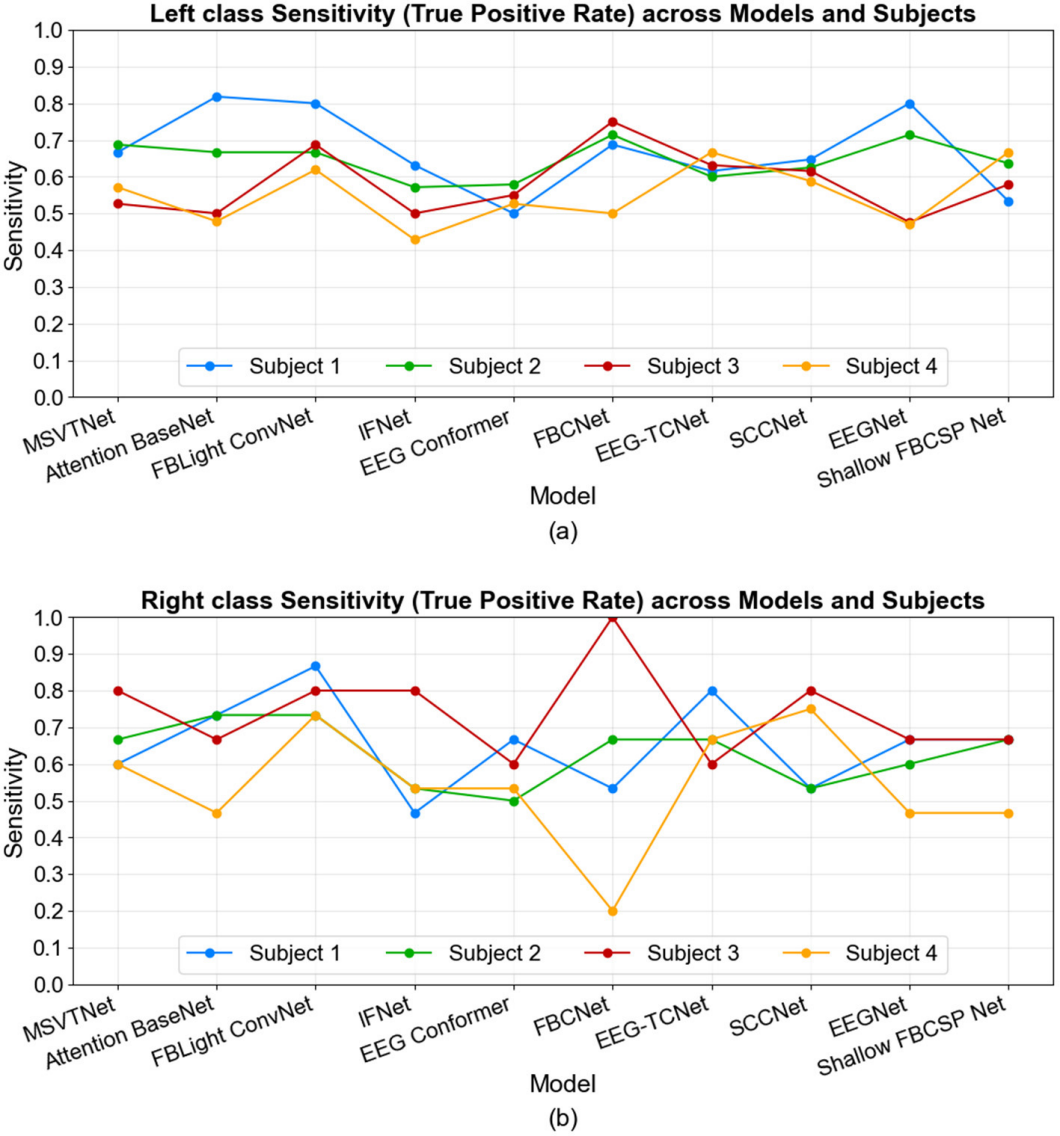
Online left **(a)** and right **(b)** classes sensitivity across models and subjects.

**Figure 8:**
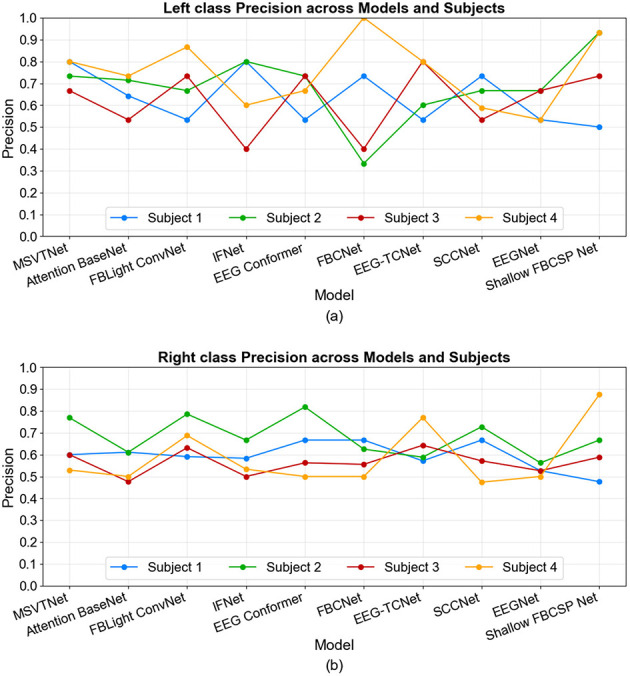
Online left **(a)** and right **(b)** classes precision across models and subjects.

The class-wise metrics in Figures 7, 8 reveal a marked precision-sensitivity trade-off that varies across decoders and participants, with no universal winner across the two MI classes. For the left class, several architectures show high and sparse precision but reduced sensitivity for certain subjects (i.e., conservative decisions that miss true left events), whereas others show the opposite pattern (liberal decisions with more false positives). For the Right class, the pattern flips across subjects, with high and sparse sensitivity but reduced precision in some subjects, indicating susceptibility to individual neurophysiology and control strategies. These patterns show that precision and sensitivity offer complementary views of performance under online BCI operation. The observed subject-wise variability provides a realistic baseline for deployment and motivates future work on adaptive calibration and thresholding to tailor decoders to individual users.

**Figure 9:**
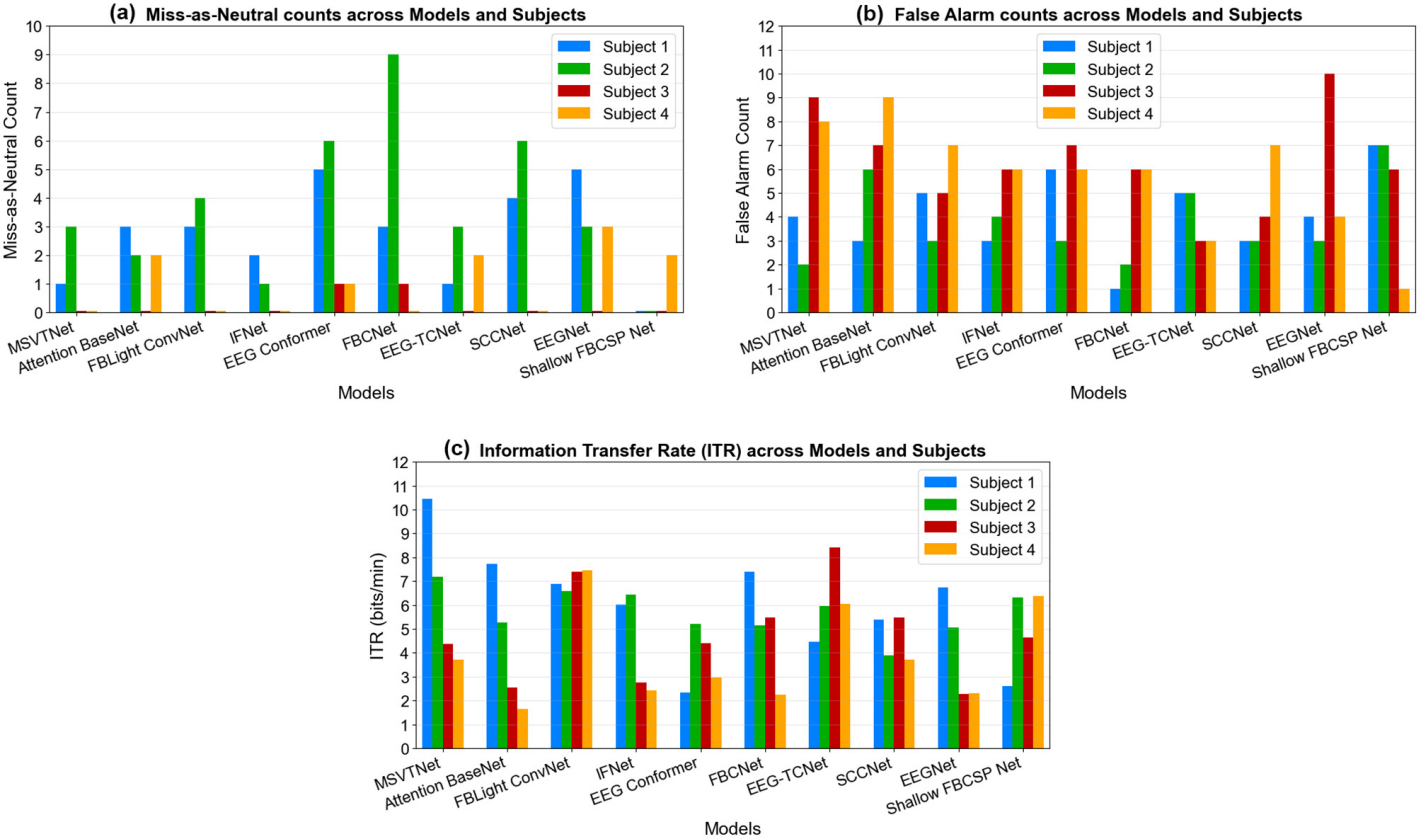
Online left and right classes miss-as-neutral rate (MANR) **(a)**, false alarm rate (FAR) **(b)**, and information transfer rate (ITR) **(c)** across selected models and subjects.

Figures 9a, b show the MANR and FAR counts for the left and right classes, respectively. The two error modes reveal complementary weaknesses of the decoders under the 3-attempt, 10-s online protocol. MANR is dominated by Subject 2 across many decoders (peaking with FBCNet and elevated for EEG-Conformer and SCCNet), indicating repeated low-confidence outputs even after multiple attempts—consistent with weak or unstable lateralized evidence for that participant. In contrast, false alarms concentrate in Subjects 3 and 4 (with pronounced counts for EEGNet, MSVTNet, and several others), i.e., decoder predictions during subjects' rest that trigger unintended cues on the screen. This subject-wise asymmetry shows that architectures are not failing uniformly: some sit at a conservative operating point (low FAR but high MANR, e.g., several decoders on Subject 2), while others are liberal (higher FAR but fewer misses, e.g., multiple decoders on Subject 3). Therefore, offline accuracy alone is insufficient; deployment must consider where each decoder lands on this miss—false-alarm frontier for each user.

### 4.2 Efficiency

Figure 9c shows the ITR results across models and participants. It can be observed that ITR magnifies the subject- and model-specific trade-offs already seen in the MANR/FAR error analyses. No single decoder dominates: MSVTNet yields the top throughput for the first participant, EEG-TCNet leads for the third participant, and FBLight ConvNet is competitive for the third and fourth. These inversions relative to offline rankings reflect that ITR integrates both accuracy and decision yield within the trial window. Decoders that were conservative for certain participants (high MANR) lose throughput despite reasonable precision and/or sensitivity (e.g., EEG Conformer and SCCNet for Subjects 1 and 2), while architectures prone to false alarms pay an accuracy penalty that similarly suppresses ITR (e.g., Attention BaseNet and EEGNet). Once more, FBLight ConvNet shows the lowest spread across participants, demonstrating stable performance from subject to subject, whereas MSVTNet shows the largest spread.

**Figure 10:**
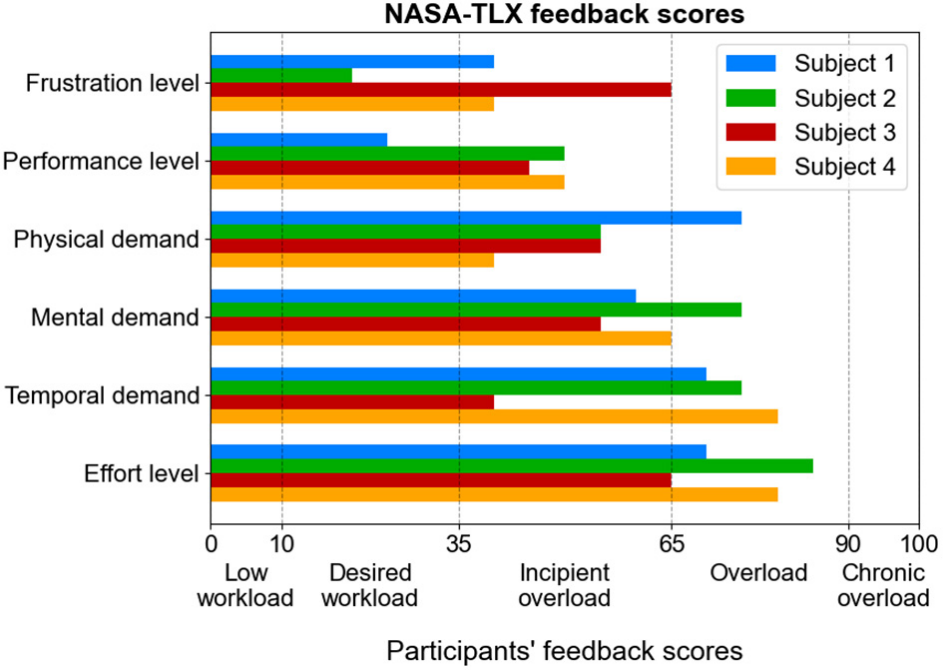
Participants' feedback scores in the NASA-TLX evaluation form.

Figure 10 shows the feedback scores reported by the participants in the NASA-TLX form. From the NASA-TLX profiles in Figure 10, Subject 1 reported strong time and physical demands, yet good performance and only modest frustration (good adaptation). Subject 2 appeared cognitively stretched but not frustrated (engaged workload). Subject 3 reported the highest frustration despite mid-level demands. Subject 4 identified time pressure and effort level as the primary sources of stress.

Overall, across subscales, the dominant burdens are temporal demand and effort, which fall in the overload band of the axis for most participants, consistent with the pressure to deliver rapid decisions in our soft real-time paradigm. Mental demand sits near the boundary between incipient overload and overload, while physical demand is generally lower (mostly in the incipient overload range), suggesting the headset and posture were tolerable. Performance ratings are comparatively favorable (lower values on this scale indicate better perceived success), and frustration remains modest overall, indicating that participants felt reasonably effective and not chronically stressed despite elevated time pressure.

### 4.3 Additional offline baselines

Table 3 benchmarks the offline accuracy of the additional 10 DL architectures under the same 2-s window protocol. Overall, their mean offline accuracies cluster in the mid-50s to low-60s (best: MSCFormer 63.43 ± 5.16%), below the top performers from Table 2 (e.g., FBLight ConvNet 70.23 ± 3.92%, FBCNet 67.57 ± 1.07%, EEG-TCNet 59.45 ± 3.33%). None of the additional models surpasses the strongest CNN/filter-bank/TCN designs already evaluated online.

**Table 3:** Offline accuracy of additional models.

**Model**	**Offline accuracy (%)**				
	**S1**	**S2**	**S3**	**S4**	**Mean** ±**SD**
TCANet	57.37	54.66	56.43	57.77	56.56 ±**1.38**
MSCFormer	58.66	61.30	63.07	70.67	**63.43** ± 5.16
CTNet	56.88	56.86	52.43	62.26	57.11 ± 4.02
EEGNeX	54.69	58.22	52.86	61.77	56.89 ± 3.94
EEG SimpleConv	58.68	60.89	57.79	56.85	58.55 ± 1.73
EEG-ITNet	50.64	53.78	48.88	49.77	50.77 ± 2.13
ATCNet	59.09	63.54	49.30	63.56	58.87 ± 6.72
EEG-inception	60.47	59.54	52.44	57.75	57.55 ± 6.72
TIDNet	38.61	35.52	45.29	44.94	41.09 ± 4.82
Deep ConvNet	48.88	52.86	47.57	50.28	49.90 ± 2.26

Bold values indicate the best results.

Transformer-hybrids (MSCFormer, CTNet) achieve mid-tier accuracy (63% and 57% on average), in line with our main finding that attention/Transformer additions do not yield a systematic advantage in the short-window regime (cf. EEG Conformer 56.68 ± 4.69% in Table 2). Lightweight CNN + TCN + attention (TCANet) shows low variance across subjects (± 1.38) but a modest mean (56.56%), suggesting stability without peak performance under a 2-s context. Older baselines (Deep ConvNet, TIDNet) underperform (≈ 50% and 41%), reinforcing the need for spectro-temporal inductive biases tailored to MI. ATCNet and EEG-inception are competitive among the added set (≈ 59% and ≈ 57.6% means) but remain below the filter-bank CNN family. Overall, the expanded offline sweep is consistent with our main findings: compact spectro-temporal CNN backbones with filter banks and lightweight temporal context remain the most reliable candidates for short-window MI decoding, while heavier attention/Transformer stacks offer, at best, incremental gains offline but are less stable online in our main experiments.

Patterns remain consistent with the selected main 10 models: Subject 4 tends to be easier offline (e.g., MSCFormer 70.67%), whereas Subjects 1–3 show more moderate scores. These subject-dependent differences mirror the inter-subject variability we observed online and underscore the value of per-subject calibration and spectral weighting.

### 4.4 Spectral analysis of offline EEG data

#### 4.4.1 Lateralization index as a function of frequency

**Figure 11:**
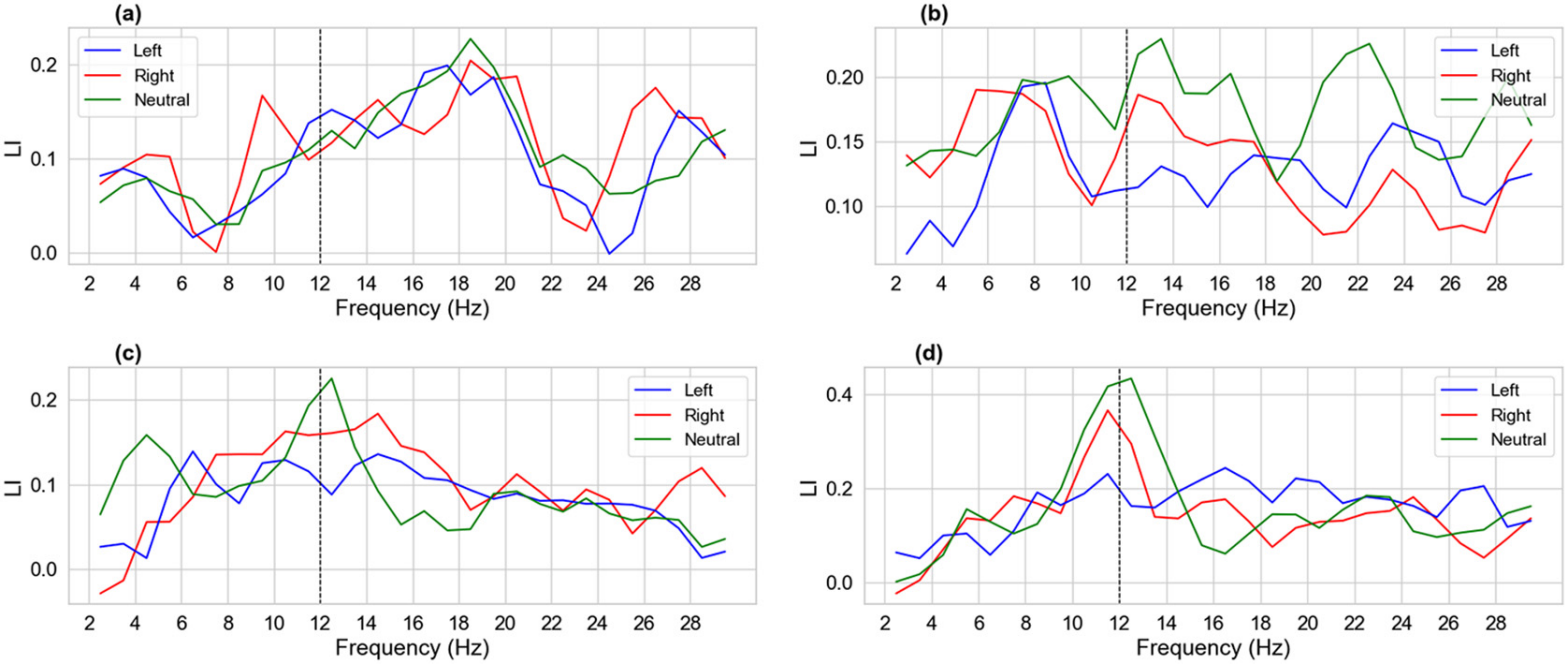
Offline Lateralization Index (LI) per class across subjects. **(a)** Subject 1—LI across frequencies (narrow 1 Hz bands). **(b)** Subject 2—LI across frequencies (narrow 1 Hz bands). **(c)** Subject 3—LI across frequencies (narrow 1 Hz bands). **(d)** Subject 4—LI across frequencies (narrow 1 Hz bands).

Figure 11 shows that the offline LI value per class across participants, computed from one-hertz PSD slices over the 2–29 Hz range, reveals subject-specific spectral asymmetries over the sensorimotor strip. Across participants, LI magnitude rises around the mu/low-beta range and then tapers (near the dashed marker at 12 Hz, around the mu peak, and immediately above), but the key determinant of decoder behavior is the class separability of LI in that band and its offset from neutral. Where left and right curves diverge from each other and from neutral around mu, decoders have more discriminative spatial–spectral information; where all three curves cluster or cross, the decision surface collapses.

Subject 1's LI for left and right rises through mu/low-beta and neutral largely tracks both (10–18 Hz), with only short windows of left–right separation (≈ 11–13 Hz). This modest MI-Neutral contrast is consistent with mid-tier online accuracies that depend strongly on decoder choice (e.g., MSVTNet/FBCNet ≈ 73% vs. others ≈ 60%–69%). Because Neutral is not cleanly isolated from MI in LI, misses and false alarms remain moderate (MANR ≈ 1–5; FAR ≈ 1–7) and class-wise precision/sensitivity trade-offs across decoders, as seen in the per-class curves. Subject 2's neutral LI is systematically high and often exceeds the MI curves over mu–beta; left and right also cross repeatedly. This weak separability explains the pronounced miss-as-neutral behavior (MANR spikes for EEG-Conformer, FBCNet, and SCCNet) and lower/variable accuracies, except for architectures that appear to down-weight ambiguous bands (MSVTNet, FBLight ≈ 66%–76% online). This ambiguity also aligns with the generally low FAR for Subject 2, as decoders are conservative when the MI vs. Neutral LI structure is unclear.

We observe for Subject 3 that around ≈ 13–18 Hz, left/right LI sit above Neutral with a relatively stable offset; outside that range (≈ 6–8 and > 20 Hz), the curves converge and/or cross. This pattern matches the better online scores for decoders capable of leveraging mu-band and low-beta separability when present (FBLight ConvNet and EEG-TCNet ≈ 73% and higher ITR), together with low MANR (MI stands out from Neutral, where the decoders are most sensitive). The elevated FAR of some architectures (e.g., EEGNet, MSVTNet) is also plausible here: when decoders weight bands where LI curves re-converge, neutral fluctuations can trigger MI outputs despite the good mu-band separability. Subject 4's neutral class shows a prominent LI peak near mu (≈ 10–12 Hz), overlapping and at times exceeding the MI curves, whereas separation improves again beyond ≈ 14 Hz. This explains the model-dependent outcomes: methods emphasizing narrow mu power alone suffer (e.g., Attention BaseNet shows higher FAR and lower online accuracy), while those exploiting broader frequency–spatial structure fare better (EEG-TCNet ≈ 75.6% online; Shallow FBCSP ≈ 77.8%), and FAR varies accordingly.

The LI analysis does not replace multichannel decoding, but it predicts the direction of online behavior: (i) clear MI–Neutral offsets in the mu/low-beta range imply higher accuracy/ITR and lower MANR; (ii) left–right crossings or neutral peaks within the mu-band imply model-dependent drops in accuracy and shifts between FAR and MANR depending on how each architecture weights the conflicted bands. This subject-specific LI structure clarifies why the same decoder can rank differently across participants and why mu-aware but broadband-robust decoders (e.g., FBLight ConvNet, EEG-TCNet) achieve competitive online performance without uniformly dominating every case.

#### 4.4.2 C3 vs. C4 mu-band power

**Figure 12:**
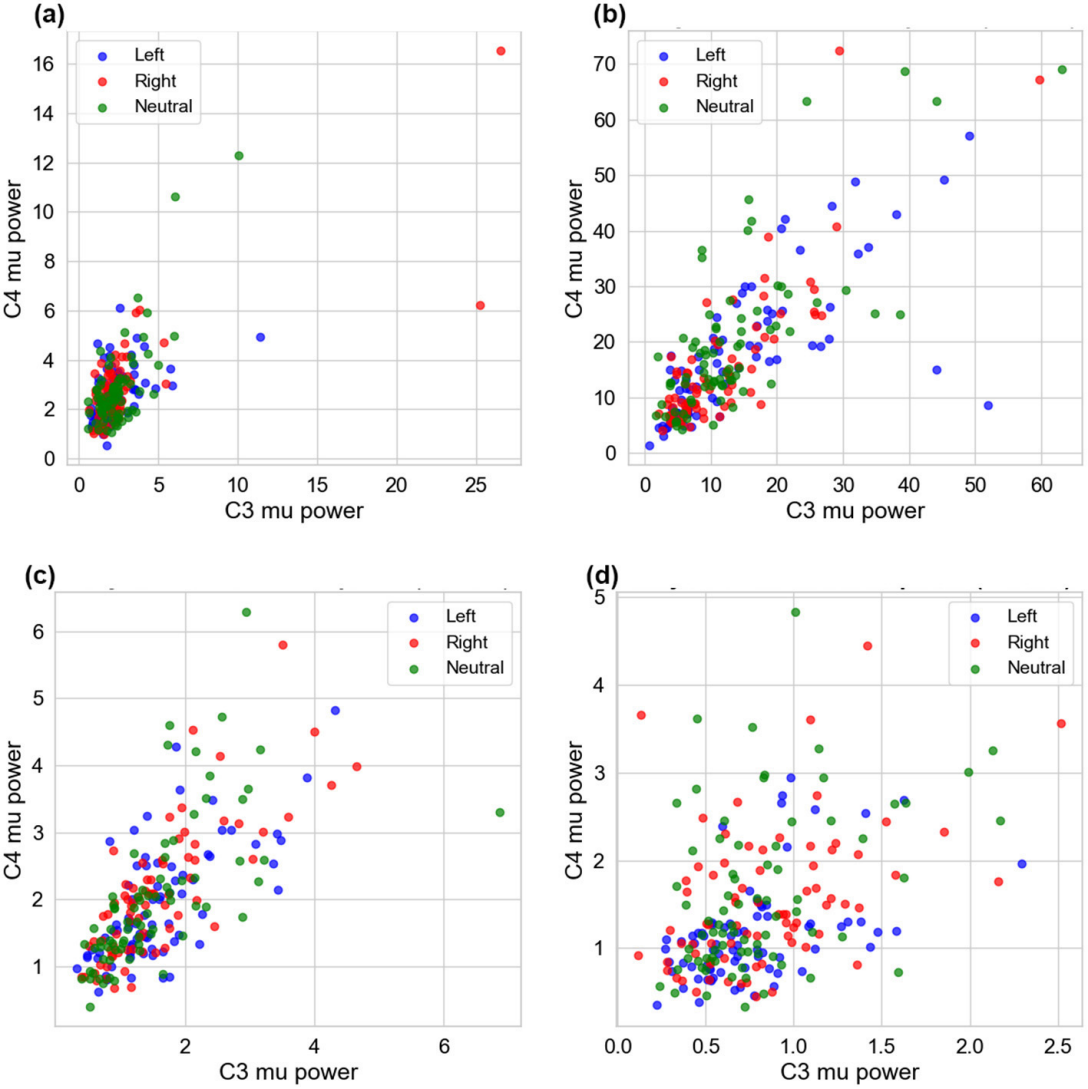
Offline C3 vs. C4 Mu-band power per class across subjects. **(a)** Subject 1–C3 vs. C4 mu power (8–12 Hz). **(b)** Subject 2–C3 vs. C4 mu power (8–12 Hz). **(c)** Subject 3–C3 vs. C4 mu power (8–12 Hz). **(d)** Subject 4–C3 vs. C4 mu power (8–12 Hz).

We visualize, in Figure 12, the trial-wise mu-band (8–12 Hz) power at the two contralateral sites for each subject. The expected pattern is weak for Subjects 1 and 4: all three classes form a compact, highly overlapping cloud with only a few outliers, indicating limited class-specific lateralization and a small modulation depth. This explains why several decoders show modest online accuracy or ITR and elevated miss-as-neutral or false-alarm rates for these participants: the features needed to differentiate the classes are simply not well expressed in mu power at C3/C4. Subject 3 shows a tighter positive correlation but with a mild tendency for left trials to sit slightly below the diagonal and right trials slightly above it; although overlap remains, this weak directional bias is consistent with the comparatively better sensitivity that some temporal/spectral CNNs achieved for Subject 3 (without implying a specific architecture is best in general). Subject 2 exhibits the broadest dynamic range (large spread along both axes) but still strong class overlap; the large variance means decoders can capitalize on power changes (supporting reasonable ITR) yet also risk class confusions if thresholds are not individualized, which matches the mixed accuracy/MANR/FAR profile observed across decoders for this subject.

#### 4.4.3 ERD/ERS topographic maps

**Figure 13:**
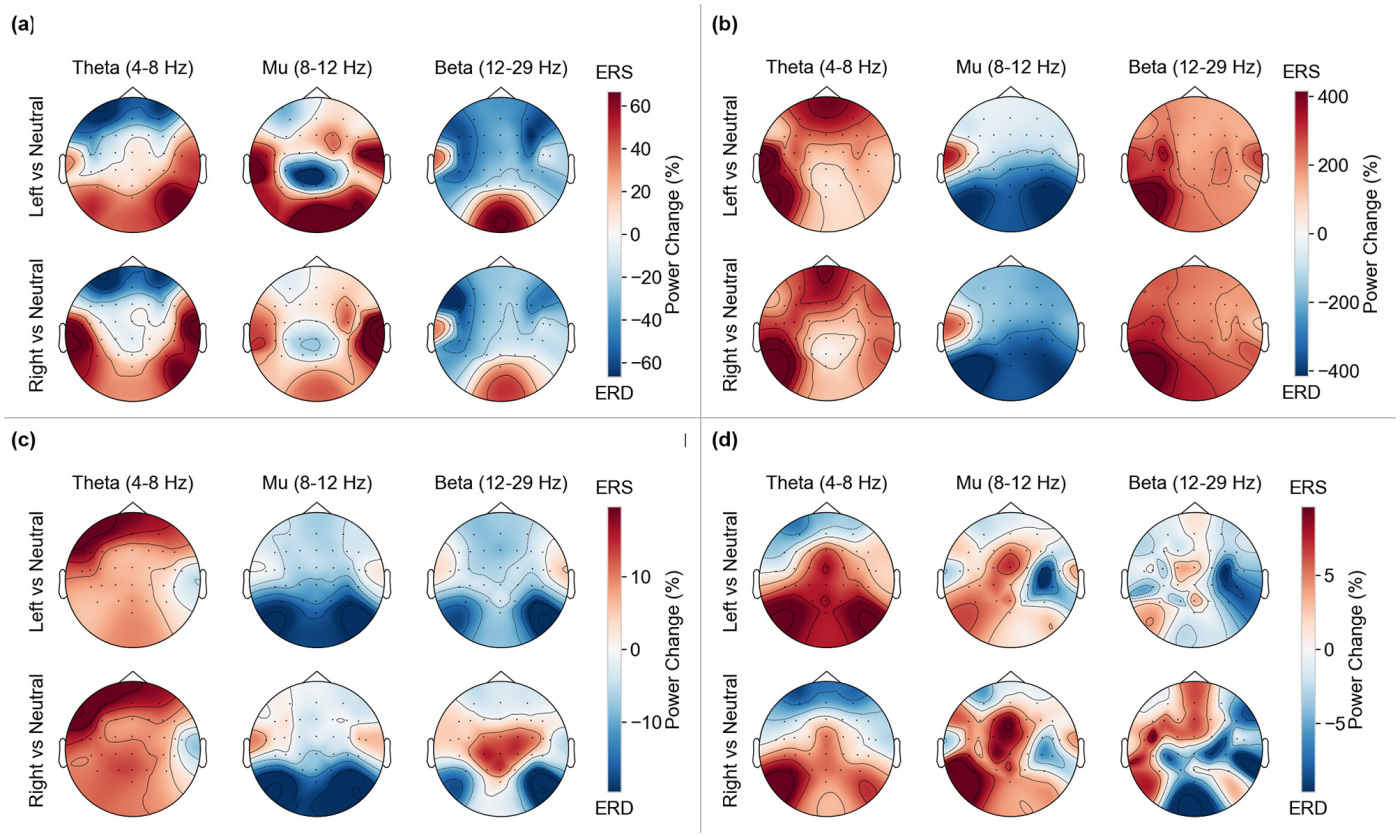
Offline percentage change in band power of MI vs. neutral across subjects. **(a)** Subject 1—percentage change in band power of motor imagery vs. neutral. **(b)** Subject 2—percentage change in band power of motor imagery vs. neutral. **(c)** Subject 3—percentage change in band power of motor imagery vs. neutral. **(d)** Subject 4—percentage change in band power of motor imagery vs. neutral.

Figure 13 shows the percent change in band power (ERD/ERS) for MI (left and right) vs. the neutral baseline in three bands: theta (4–8 Hz), mu (8–12 Hz), and beta (12–29 Hz). When read against the obtained online metrics, the topographic maps reveal several consistent patterns. Subject 1 (Figure 13a) shows clear central mu-ERD (blue) for both left and right—more pronounced for the left—with weak lateralization (bilateral behavior) in accordance with Figure 12a. Nevertheless, these sensorimotor patterns are relatively clean, class-separable, and line up with Subject 1's satisfactory online accuracy across many decoders and moderate FAR/MANR (neither extreme).

Conversely, Subject 2 (Figure 13b) exhibits a strongly bilateral mu-ERD in line with Figure 11b, although broad and similar for left and right, spanning central sensors for both left and right, and widespread beta-ERS/ERD of the same sign for the two classes. This bilateral behavior means there is no reliable left-right flip nor strong contrast, thereby explaining why decoders tend to abstain (higher MANR observed for Subject 2): the MI-vs.-Neutral contrast is large (hence big percent changes), but left vs. right evidence is weak, so the decoders often abstain (the screen stays black).

Subject 3 (Figure 13c) shows fairly midline/bilateral mu-ERD and a more mixed beta pattern (right vs. neutral has centro-parietal beta-ERS, while left shows beta-ERD), which makes class evidence decoder-dependent, consistent with Subject 3's spread in precision/sensitivity. This behavior can confuse decoders that rely on a fixed contralateral prior and explains the higher FAR observed for some decoders that overweight beta activity.

Subject 4 (Figure 13d) has an overall smaller ERD/ERS percent change (note the tighter color scale), although it is clearly contralateral for left MI, which produces a strong right-central mu-ERD focus. This aligns with the observation that some decoders for Subject 4 incur a FAR penalty (spurious arrows) unless they are strongly regularized or employ robust spatial filtering. Conversely, filter-bank Common Spatial Patterns (CSP)-style approaches handled Subject 4 comparatively well, as they maximize inter-class differences despite the overall smaller ERD/ERS change relative to the neutral baseline, thereby matching the strong online result observed for Shallow FBCSP.

## 5 Practical recommendations for decoder design

### 5.1 From the effectiveness

The accuracy patterns—ranking inversions between offline and online and a sizable subject-to-subject spread—argue for decoders engineered first for online robustness rather than offline peaks. Concretely, favor compact spectro-temporal CNN backbones with dilated temporal kernels or lightweight temporal convolutional network (TCN) blocks, which exhibit good online stability over heavy attention stacks; enforce strong spatial lateralization (e.g., around C3/C4) to curb left-right leakage; and control capacity with depthwise separable layers and regularization to resist overfitting to curated trials. Train with a curriculum from longer to brief windows, plus augmentations that mimic online noise or idle segments. Because variability across users is substantial, include rapid per-subject calibration (few trials for threshold or temperature tuning) and report online mean and variance alongside first-attempt vs. cumulative success, ITR, MANR, FAR, and latency. Designs that minimize online variance across subjects—not just maximize mean accuracy—are the most promising for practical BCI deployment.

Sensitivity and precision results argue for calibrated, subject-aware operating points. Concretely, the following enhancements could be applied to improve the results: (i) tune class-wise decision thresholds (or temperature-scaled probabilities) per subject to target a balanced precision-sensitivity or task-specific cost; (ii) apply temporal smoothing or abstention to suppress transient left-right flips that inflate false positives; (iii) encourage lateralized spatial evidence (stronger weighting around contralateral motor areas) to reduce cross-class leakage; and (iv) use class-balanced or focal losses to stabilize precision/recall when class priors or discriminability differ across users. Brief per-subject adaptation (e.g., few-shot fine-tuning or *post-hoc* calibration during a short calibration run) is likely to convert several borderline precision or sensitivity profiles into higher ones while preserving real-time constraints.

The results of MANR and FAR highlight the need for practical solutions. First, adopt subject-specific calibration of class thresholds (or temperature scaling) to move each decoder toward a balanced point by prioritizing FAR control when safety is critical or MANR reduction when responsiveness is paramount. Second, add temporal safeguards such as brief dwell times, refractory periods after an activation, and confidence-weighted smoothing or abstention; these typically help reduce FAR with minimal impact on time to correct within the allowed attempts. Third, strengthen neutral-class modeling (e.g., an explicit neutral detector, artifact gating for ocular or EMG bursts, and lateralization-focused spatial filters around contralateral motor areas) to reduce spurious activations while preserving motor-class detectability.

### 5.2 From the efficiency

Operating points should be chosen to maximize expected ITR, not accuracy alone. Practically, this means per-user calibration of class thresholds or temperature scaling, confidence-weighted early stopping within the multi-attempt window (commit when confidence is high; otherwise abstain and re-acquire), and short refractory or dwell periods to curb false alarms without starving selections. Strengthening idle modeling and emphasizing lateralized spatial evidence can simultaneously reduce false activations and misses, converting more attempts into valid selections. Finally, analyzing the first attempt ITR alongside the cumulative ITR (best of attempts within the window) will disentangle decoder quality from human-in-the-loop adaptation and guide the choice of architecture and operating point for each user.

From the NASA-TLX feedback scores reported by the participants, the design implications are clear: reduce temporal pressure through adaptive pacing or confirm-to-act schemes, optimize decoder design to reduce the required mental effort, smooth classifier outputs, allow abstention to limit error-induced effort, personalize training and feedback, and continue ergonomic refinements to keep physical demand low while maintaining responsiveness.

### 5.3 From the offline EEG spectral analysis

Two implications follow from the LI analysis. First, favor baseline-normalized asymmetry over raw power: using ΔLI = LI_MI_−LI_Neutral_ (or ERD percentage change at C3/C4) suppresses tonic inter-hemispheric bias and highlights task-evoked desynchronization. Second, use subject-specific spectral weighting: learn or set sub-band masks that up-weight upper-mu/low-beta where separability is present (e.g., Subject 3), down-weight neutral-dominated bands (as seen in Subject 2 and Subject 4), and encourage left/right to carry opposite-signed lateralization in informative bands. In practice, this can be implemented with (a) filter-bank CNN branches plus per-subject gates, (b) frequency-attention constrained to favor contralateral C3/C4 evidence, or (c) a brief calibration that estimates ΔLI peaks and sets class-wise priors/thresholds accordingly. Aligned to these LI profiles, decoders should reduce left–right swaps, lower miss-as-neutral for neutral-dominant users and improve online stability without adding latency.

Offline C3 vs. C4 mu-band power suggests designing opposite-signed evidence for left vs. right without hard-coding CSP. This can be achieved through adding a lightweight lateralization head during calibration by learning a linear boundary in the (C3-mu, C4-mu) plane—or in LI = (C4 − C3)/(C4+C3)—and using it as an auxiliary loss/feature for the DL decoder. This should be completed with normalization against neutral by computing Δμ = μ(MI)−μ(Neutral) (or ΔLI) per session to suppress tonic asymmetries. These neutral-referenced features can then be used for thresholding and early stopping. Robustness to weak lateralizers (Subject 1-like) could be achieved by combining mu asymmetry with complementary cues (upper-beta desynchronization, transient ERD timing, broader sensorimotor cluster) and by applying temporal smoothing to decisions to reduce spurious flips. Together, these steps link the scatterplot evidence to actionable decoder design: neutral-referenced and subject-tuned lateralization features to reduce miss-as-neutral and false alarms.

ERD/ERS results suggest that training decoders on MI vs. neutral contrasts improves performance, not on absolute band power. ERD/ERS maps confirm that neutral-relative baselining isolates task-evoked changes. Several failure modes (e.g., Subject 2 symmetry and Subject 4 low amplitude) are invisible in absolute power. Incorporate neutral-relative losses into the performance objective, which should reduce MANR (clearer MI evidence) and FAR (less tonic drift). Additionally, subject-specific spectral-spatial weighting should be considered. For users like Subject 2, emphasizing lateralization could be achieved by penalizing bilateral mu-ERD and up-weighting contralateral channels. For users like Subject 3, beta features could be allowed but with class-consistent signs (beta-ERS for one class, ERD for the other) to avoid model-dependent flips. For users like Subject 4, filter-bank density and spatial regularization could be increased to harvest weak yet stable patterns. Concretely, the following interventions may be undertaken: (i) Consider adding a filter-bank branch with per-subject gates learned during calibration; (ii) include a C3/C4 lateralization prior as a contrastive loss encouraging opposite signs for left vs. right in the informative bands; (iii) pick the operating point per subject to balance precision vs. sensitivity based on ERD strength (e.g., more conservative thresholds for low-SNR users to cut FAR and more liberal for symmetric users to cut MANR). These steps align the decoder's inductive bias with the subject-specific physiology, which should yield gains in accuracy/ITR while reducing misses and false alarms in online use.

## 6 Limitations and future work

The present study has some limitations that qualify the interpretation of our findings and pave the way for future work on this topic.

- Small cohort: only four participants were involved in this study. This limits statistical power and generalizability, considering the high inter-subject variability of ERD/ERS physiological behavior. Therefore, our results should be read as a controlled case-series benchmark (four subjects), focused on within-subject online behavior under 2-s windows; we therefore refrain from across-subject hypothesis testing and limit claims to subject-wise results. Future research should perform in-depth experiments with a broader population.
- Task scope: we evaluated two MI classes (left/right) along with a neutral baseline. Future research should assess how performance and error modes may differ across additional MI classes or in hybrid continuous control.
- Short time windows and soft real-time: in our experiment, we used fixed 2-s epochs within a soft real-time loop. Future work should assess how operating characteristics change under shorter window lengths, sliding windows, adaptive dwell times, or hard real-time constraints.
- Session depth: each subject completed a limited number of online sessions; practice, fatigue, and user–decoder co-adaptation were not modeled. Multi-day studies are needed in future work to quantify within-subject drift over long periods and across different physiological or emotional states.
- Hardware and montage specifics: our results were obtained using a 32-channel sensorimotor-centric montage at 256 Hz. Low-density caps, dry electrodes, or different sampling rates remain to be tested.
- Model training and hyperparameters: ten representative DL decoders were trained with their respective default parameters to keep comparisons fair. Future studies should assess whether per-subject hyperparameters tuning may result in a reduced performance gap between architectures, especially with lightweight architectures.
- Operating-point calibration: thresholds, smoothing, and abstention were kept simple and largely uniform to reveal raw model behavior. Different per-subject operating points could balance false alarms for miss-as-neutral and affect ITR in future studies.
- Participants health and context: healthy users in laboratory settings may not reflect clinical or at-home deployments. Further studies are needed to assess the usability of DL decoders in clinical or at-home settings.

## 7 Conclusion

This study assessed representative DL EEG decoders under a soft real-time, closed-loop paradigm with brief temporal windows. By reporting class-wise sensitivity and precision, along with miss-as-neutral, false-alarm rate, and information-transfer rate, we demonstrated that offline rankings do not directly translate to interactive use and that subject-to-subject variability is substantial. Topographic ERD/ERS analysis revealed that bilateral or neutral-dominated patterns produced elevated MANR, while mixed or beta-weighted patterns increased FAR for decoders that over-weight beta. Neutral was comparatively stable, but the dominant error mode across decoders remained lateral swaps between left and right, underscoring the importance of operating point selection (thresholds, smoothing, abstention) as much as architectural choice. Compact spectro-temporal CNN backbones with temporal kernels and lightweight TCN block designs tended to preserve performance more consistently online, whereas heavier attention stacks were more sensitive to subject shift and session dynamics.

Future studies on DL EEG decoders should focus on online usability rather than only on offline scores validated on publicly available datasets. Concretely, decoders should target short-window stability, low latency, and calibrated confidence; adopt baseline-normalized features (MI vs. Neutral ERD %) and/or ΔLI = LI_MI_−LI_Neutral_ to suppress tonic asymmetries; add subject-specific spectral weighting by up-weighting informative mu/low-beta sub-bands and down-weighting neutral-dominated bands; and pair compact spectro-temporal backbones with lightweight temporal context (dilations or TCN) and uncertainty-aware decision rules. Training should include curricula spanning longer to shorter windows and augmentations that mimic streaming noise and idle periods. Evaluation should occur in a closed loop and report ITR, MANR, FAR, latency, and user-level variability, with rapid per-subject calibration protocols. Releasing streaming evaluation code and prioritizing low user variance—not just higher offline accuracy—while targeting cross-device generalization and energy-constrained on-device inference will move the field toward deployable, real-time BCI.

## Data Availability

The raw data supporting the conclusions of this article will be made available by the authors upon request.
